# Long-Term Dexamethasone Exposure Down-Regulates Hepatic TFR1 and Reduces Liver Iron Concentration in Rats

**DOI:** 10.3390/nu9060617

**Published:** 2017-06-17

**Authors:** Huifang Li, Shuxia Jiang, Chun Yang, Shu Yang, Bin He, Wenqiang Ma, Ruqian Zhao

**Affiliations:** Key Laboratory of Animal Physiology and Biochemistry, College of Veterinary Medicine, Agricultural University, Nanjing 210095, China; 2014107015@njau.edu.cn (H.L.); 2014107016@njau.edu.cn (S.J.); 2015107016@njau.edu.cn (C.Y.); 2014207008@njau.edu.cn (S.Y.); heb@njau.edu.cn (B.H.); zhaoruqian@njau.edu.cn (R.Z.)

**Keywords:** dexamethasone, liver iron content, TFR1, rat

## Abstract

Exposure to stress is known to cause hepatic iron dysregulation, but the relationship between prolonged stress and liver iron metabolism is not yet fully understood. Thirty 13-week-old female Sprague–Dawley rats were randomly divided into two groups, as follows: the control group (saline-injection) and the dexamethasone group (Dexamethasone (Dex)-injection 0.1 mg/kg/day). After the 21-day stress trial, the results showed that chronic Dex administration not only impaired serum corticosterone (*p* = 0.00) and interleukin-6 (IL-6) (*p* = 0.01) levels, but also decreased white blood cell counts (*p* = 0.00), and reduced blood lymphocyte counts (*p* = 0.00). The daily Dex-injection also significantly reduced body weight (*p* < 0.01) by inhibiting food intake. Consecutive Dex administration resulted in decreased iron intake (*p* = 0.00), enhanced serum iron levels (*p* = 0.01), and increased the serum souble transferrin receptor (sTfR) content (*p* = 0.00) in rats. Meanwhile, long-term Dex exposure down-regulated duodenal cytochrome b (DCYTB) (*p* = 0.00) and the divalent metal transporter 1 (DMT1) (*p* = 0.04) protein expression, but up-regulated ferroportin (FPN) protein expression (*p* = 0.04). Chronic Dex administration reduced liver iron concentration (*p* = 0.02) in rats. Hepatic transferrin receptor 1 (TFR1) expression was lowered at the protein level (*p* = 0.03), yet with uncoupled mRNA abundance in Dex-treated rats. Enhanced iron-regulatory protein (IRP)/iron-responsive element (IRE) binding activity was observed, but did not line up with lowered hepatic TFR1 protein expression. This study indicates that long-term Dex exposure reduces liver iron content, which is closely associated with down-regulated hepatic TFR1 protein expression.

## 1. Introduction

Iron is an essential element in such vital functions as oxygen delivery, energy production, protein synthesis, DNA replication and cellular respiration. It has been proven that the alteration of iron homeostasis underlies a variety of pathological conditions in mammals [1]. Iron deficiency results in reduced oxygen transport, diminished activity of iron-dependent enzymes, poor growth development and cognitive skills [2,3]. Chronic iron overload could cause oxidative stress and increase the risk of liver cirrhosis, cancer, and neurodegenerative diseases [4]. Therefore, maintenance of systemic and cellular iron homeostasis is vital for normal physiological functions in mammals.

Iron homeostasis is maintained by two regulatory systems: hepcidin/ferroportin, and iron-regulatory protein (IRP)/iron-responsive element (IRE), to ensure appropriate supplies [5]. Systemic iron homeostasis is controlled by hepcidin, which acts as a negative regulator of iron release by degradation of the iron exporter ferroportin, from enterocytes, hepatocytes or macrophages [6,7,8]. Cellular iron homeostasis is primarily regulated by IRP1 and IRP2 in mammals. IRP1/2 binds to IREs in the 3′ or 5′ UTR of mRNAs encoding for important proteins of iron uptake (transferrin receptor 1 (*Tfr1*) and divalent metal transporter 1 (*Dmt1*), storage (ferritin) and export (ferroportin) to regulate cellular iron metabolism [9,10]. Balanced iron uptake, utilization, storage and export are strictly regulated at both systemic and cellular levels to keep iron homeostasis.

Exposure to uncontrollable and unpredictable stressors is a common daily occurrence in the lifestyle of modern society [11]. It is reported that dysregulation of iron metabolism and storage, could be caused by stress [12,13,14,15,16]. Recent advances in stress research have revealed that psychological stress decreases blood iron levels [12,13,14], and induces hepatic iron accumulation in vivo and in vitro [14,15,16]. However, the impact of stress is highly controlled by the types (psychological vs. physiological vs. physical), intensity, and duration of the stressors [17,18]. It is noted that chronic mild stress elevates interleukin-6 (IL-6) to stimulate hepcidin expression in rats [6,19]. Therefore, glucocorticoid exposure-induced immunosuppression associated with decreased IL-6, could play a role in iron metabolism, via inhibition of hepcidin synthesis [20]. To our knowledge, little is known about the effect of prolonged stress induced immunosuppression on iron metabolism.

The physiological stress responses to stressful stimuli induces the activation of the hypothalamic-pituitary-adrenal axis and releases the glucocorticoids (cortisol in humans; corticosterone in rodents) from the adrenal cortex [21,22]. Dexamethasone, a synthetic glucocorticoid with high-affinity, has been applied to induce stress response and immunosuppression [23,24,25]. Therefore, the aim of the present study is to examine the effect of prolonged stress (21-day dexamethasone (Dex) administration) on iron metabolism and explore the possible mechanism in rats.

## 2. Materials and Methods

### 2.1. Animals and Experimental Design

Thirty 13-week-old female Sprague–Dawley rats weighing 320–340 g were randomly divided into two groups of fifteen rats each, as follows: the control group (saline-injection, Con) and the dexamethasone group (Dex-injection, Dex). A once-daily injection of Dex (water-soluble dexamethasone; D4902, Sigma, San Francisco, CA, USA) was administered for 21 days at the dose of 0.1 mg/kg/day via intraperitoneal injection. The dexamethasone dose was selected according to the previous study of Jahng et al. (2008) [23]. Rats were housed individually in standard cages, in a room with controlled temperature (22 ± 2 °C) and humidity (55% ± 15%), on 12-h light/dark cycles. A standard AIN-93G diet and deionized water were provided *ad libitum* to each cage. All rats were obtained and raised in the Laboratory Animal Research Center of Jiangsu University, Zhenjiang, China. The experiment was carried out following the guidelines of the Animal Ethics Committee of Nanjing Agricultural University.

### 2.2. Data and Sample Collection

Both body weight and feed consumption were monitored at 2-day intervals for 21 days. All rats were deeply anesthetized by intraperitoneal injection of 7% chloral hydrate and killed humanely at the end of experiment. Blood samples were collected from the aorta abdominalis using EDTA-2K coated tubes for hematological tests. Serum samples were obtained from blood by centrifuging at 3000 g for 10 min, and stored at −80 °C for further use. The fresh tissues (duodenum and liver, spleen, kidney, dorsal muscles) were immediately removed and snap frozen in liquid nitrogen, and kept in a −80 °C freezer until analysis.

### 2.3. Histological Analysis of Liver

Briefly, liver specimens were first fixed in a 4% formaldehyde-buffered solution for 24 h and processed with paraffin. The blocks were subsequently sectioned at 5 µm on the longitudinal plane and stained using the Mallory method of Prussian blue staining [26].

### 2.4. Haematological Parameters and Serum Concentration of Corticosterone, IL-6 and Iron Parameters

Hemocytes were detected by Automated Hematology Analyzer (BC-2800, Mindray, Shenzhen, China). Serum corticosterone was assayed by a Corticosterone ELISA kit (12021511C, Enzo Life Sciences, New York, NY, USA). The IL-6 level in the serum was tested using the enzyme immunoassay (340354, R&D Systems, Minnesota, MN, USA). Ferritin (SEA518Ra, Cloud-clone Corp, Houston, TX, USA) and souble transferrin receptor (sTfR; F15186-A, Feiya Biological Technology Company, Nanjing, China) were quantified using the ELISA kits, respectively. Serum iron (6063-2012, Shino-Test Corporation, Tokyo, Japan), unsaturated iron-binding capacity (UIBC) (6062-2012, Shino-Test Corporation, Tokyo, Japan), and transferrin (0333-2012, LEADMAN, Beijing, China) were measured by an automatic analyzer (7020, Hitachi High-Tech Crop., Tokyo, Japan) with commercial kits. All kits were used following the manufacturers’ instructions. Total iron binding capacity (TIBC) is equivalent to the sum of UIBC and blood iron. Transferrin saturation (TS) is calculated by dividing serum iron by TIBC value.

### 2.5. Iron Measurement in Tissues

Digestion of samples was conducted using the electric heating method, according to previous study [27]. Exactly 0.5 g of liver, duodenum, spleen, kidney and dorsal muscles were weighed and digested with 10 mL HNO_3_:HClO_4_ (8:2 mL) acid mixture in a 50 mL glass flask. The digestion conditions in the microwave digestion system (EHD36 electrothermal hotblock digester, Labtech, Boston, MA, USA) followed the sequence of 30 min at 90 °C; 30 min at 120 °C; 120 min at 160 °C; and 180 °C, until about 2 mL residue was left; then cooled for 10 min. The resulting solutions were diluted to a final volume of 50 mL. Iron concentrations in the liver were determined by the Graphite Atomic Absorption Spectrometer (Z-2000, Hitachi High-Tech, Tokyo, Japan).

### 2.6. RNA Isolation and Quantitative Real-Time PCR

Total RNA was isolated from duodenum (60 mg) and liver samples (40 mg) with 1 mL TRIzol reagent (15596026, Invitrogen, Carlsbad, CA, USA), according to the manufacturer’s instructions. A total of 2 µg of RNA was treated with RNase-free DNase and reverse-transcribed to cDNA by PrimeScript^®^ 1st Strand cDNA Synthesis Kit (D6110A, TaKaRa, Dalian, China). A total of 2 µL of diluted cDNA (1:25, vol/vol) was used as a template in PCR reactions on a real-time PCR system (Mx3000P, Stratagene, La Jolla, CA, USA). All the primers for real-time PCR were synthesized by Generay Biotech., China and listed in Table 1. The 2^−ΔΔCt^ method was used to analyze real-time PCR data [28]. Acidic ribosomal phosphoprotein P0 (Arbp) was used as an internal control for normalizing.

### 2.7. Total Protein Extractions and Western Blotting Analysis

Briefly, the total protein was extracted from 60 mg of frozen duodenum and liver samples by RIPA buffer containing the protease inhibitor (04906837001, Roche Diagnostics, Mannheim, Germany). Protein concentration was measured with a Pierce BCA Protein Assay kit (23225, Thermo Scientific, Waltham, MA, USA). Liver hepcidin was assayed by a hepcidin ELISA kit (H252, Jiancheng Bioengineering Institute, Nanjing, China). Western blot was carried out according to the manufacturer’s instruction (Bio-Rad, Hercules, CA, USA). The following primary antibodies were used: DCYTB (SAB2700288, Sigma, Kawasaki-shi, Japan, 1:1000), DMT1 (ab55733, Abcam, Cambridge, UK, 1:500), FTH (BS6175, Bioworld, Visalia, CA, USA, 1:1000), FTL (ab69090, Abcam, Cambridge, UK, 1:1000), FPN (sc-49668, Santa Cruz, Dallas, TX, USA, 1:1000), STAT5 (BS2427, 1:1000, Bioworld, Visalia, CA, USA), TF (HPA001527, Sigma, Kawasaki-shi, Japan, 1:1000), TFR1 (10084-2-AP, Proteintech, Manchester, UK, 1:1000), TFR2 (SAB270078, Sigma, Kawasaki-shi, Japan, 1:1000), ZIP14 (ab123988, Abcam, Cambridge, UK, 1:500), IRP1(BS1761, Bioworld, Visalia, CA, USA, 1:1000), IRP2 (ab181153, Abcam, Cambridge, UK, 1:1000). The β-actin (AP0060, Bioworld, Visalia, CA, USA, 1:10,000) and tubulin-a (BS1966, Bioworld, Visalia, CA, USA, 1:10,000) were used as loading control. The area and density of protein bands were captured and imaged by VersaDoc 4000MP system (Bio-Rad, Hercules, CA, USA) and analyzed by Quantity One software (Bio-Rad, Hercules, CA, USA).

### 2.8. RNA Electrophoretic Mobility Shift Assay (REMSA)

The total protein was extracted from 40 mg liver samples by RIPA buffer containing the protease inhibitor. For the Electrophoretic Mobility Shift Assay (EMSA), 20 µL reactions contain: Nuclease-Free Water, 10 × REMSA Binding Buffer, 500 mM DTT, 50% glycerol, tRNA (20158, Thermo Scientific, Waltham, MA, USA), liver protein extracts, biotinylated *Tfr1* probe and unlabeled *Tfr1* probe. Biotinylated *Tfr1* probe 5′-UAUUUAUCAGUGACAGAGUUCACUAUAAAUA-3′-Biotn and unlabeled *Tfr1* probe 5′-UAUUUAUCAGUGACAGAGUUCACUAUAAAUA-3′ were synthesized by Genpharma Biotech., China. Biotinylated RNAs were always added last and the reactions were incubated for 20 min at room temperature. Afterward, the samples were carefully mixed with 5 μL of 5 × REMSA Loading Buffer, pre-electrophorese of the 4% polyacrylamide gel, for 60 min in 100 V at 4 °C, then 20 μL mixed reactions were loaded on a gel for 60 min, positively charged nylon membranes were conducted by tank blotting in 0.5 × TBE at 35 V for 40 min at 4 °C, and crosslinked at 120 mJ/cm^2^ at 254 nm for 4 min. The Chemiluminescent Nucleic Acid Detection Module (20158, Thermo Scientific, Waltham, MA, USA) was used for detecting RNA-binding activity according to the manufacturer’s instruction.

### 2.9. Statistical Analysis

All data are presented as means ± SEM. The data were tested for normal distribution, and statistical significance was performed using One-way ANOVA in SPSS 20.0 for windows. A value *p* < 0.05 was considered statistically significant.

## 3. Results

### 3.1. Serum Corticosterone, IL-6 and Blood Parameters

**Figure 1:**
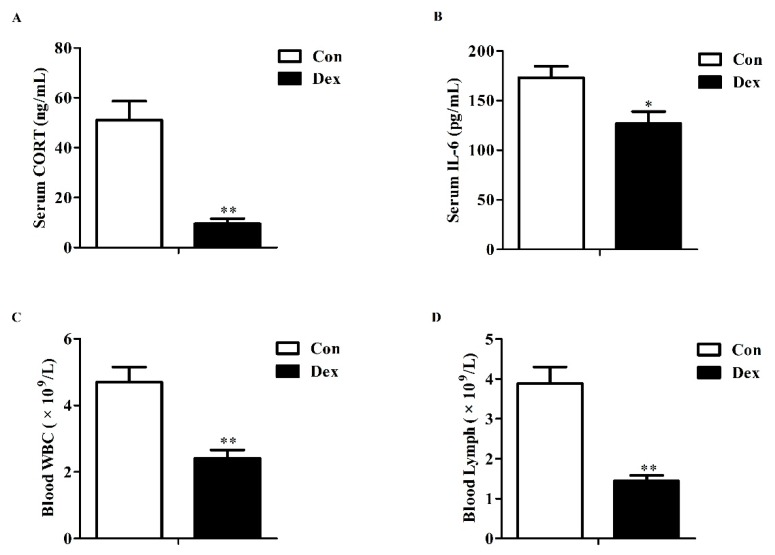
Effects of dexamethasone (Dex) administration on serum CORT, IL-6 levels and hemocytes in rats. (**A**) Dex administration lowers serum CORT level. Lowered CORT level is caused by the feedback inhibition of the HPA axis response to long-term Dex exposure; (**B**) Long-term Dex exposure reduces serum IL-6 level; (**C**) Dex administration decreases blood WBC counts; (**D**) Long-term Dex exposure declines Lymph counts. Definitions: CORT, corticosterone; IL-6, interleukin-6; WBC, white blood cell; Lymph, lymphocyte. Values are expressed as Mean ± SEM, *n* = 10, different from control, * *p* < 0.05 and ** *p* < 0.01.

Long-term Dex exposure resulted in a significant reduction in serum corticosterone (*p* = 0.00, Figure 1A), IL-6 concentrations (*p* = 0.01, Figure 1B), white blood cell (*p* = 0.00, Figure 1C) and lymphocyte counts (*p* = 0.00, Figure 1D), which were in line with synonyms of immunosuppression.

### 3.2. Body Weight, Food Intake and Iron Intake

**Figure 2:**
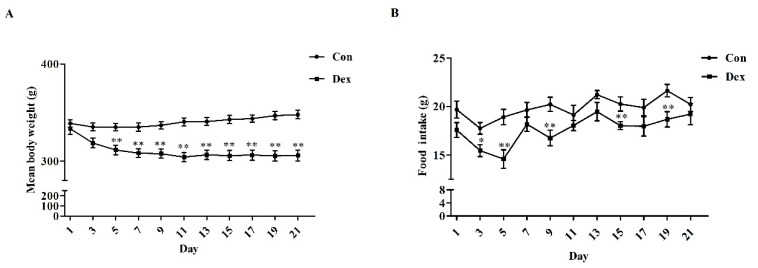

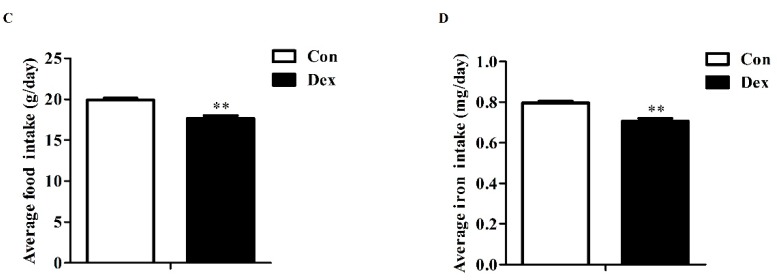
Dex administration inhibits growth, food intake and iron intake in rats. (**A**) Effect of long-term Dex exposure on growth rate; (**B**) Effect of Dex administration on food intake; (**C**) Average food intake in 21 days; (**D**) Average iron intake in 21 days. Body weight and food intake were monitored every two days. Average iron intake was calculated on the basis of average food intake and iron content of food. Definitions: Con, control; Dex, dexamethasone. Values are means ± SEM, *n* = 15, different from control, * *p* < 0.05 and ** *p* < 0.01.

Daily Dex-injections significantly reduced body weight (Figure 2A), average food intake (*p* = 0.00, Figure 2C) and average iron intake (*p* = 0.00, Figure 2D). For body weight, the remarkable inhibition was noted from the fifth day to the end of the experiment. In addition, the analysis of food intake revealed a reduction over 21 days as opposed to the control group (Figure 2B, *p* = 0.01 on day 3; *p* < 0.01 on days 5, 9, 15, 19).

### 3.3. Serum Iron Parameters

Table 2 lists the effects of Dex administration on serum iron parameters in rats. Compared with the control group, the Dex-injection greatly elevated serum iron levels (*p* = 0.01) and serum soluble transferrin receptor (sTfR, *p* = 0.03), while having no obvious effect on TIBC, UIBC, transferrin, transferrin saturation and ferritin.

### 3.4. Duodenal Expression of Iron Metabolism-Related Genes

**Figure 3:**
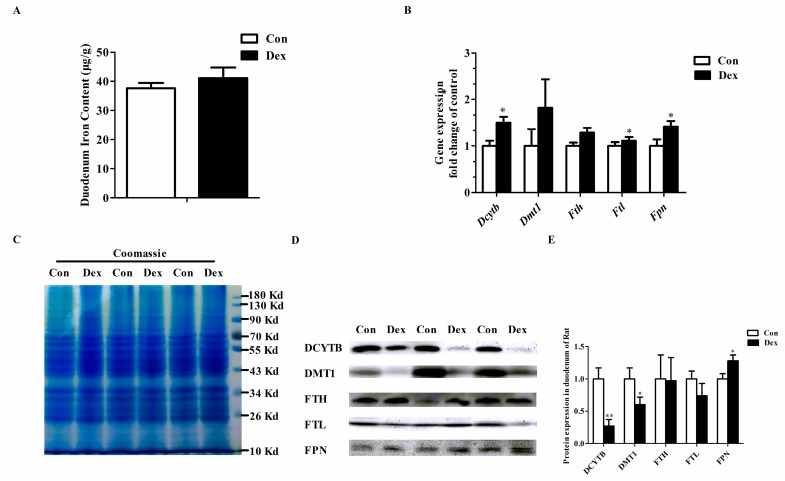
Dex administration alters the duodenal expression of genes for iron metabolism in rats. (**A**) Duodenum iron content in the two groups; (**B**) Comparisons of iron metabolism genes expression in the duodenum between the two groups; (**C**) Coomassie brilliant blue (CBB) staining confirms equal protein loading of duodenal samples; (**D**) Western blot bands of duodenal iron-metabolism genes; (**E**) Duodenal protein expression of iron metabolism-related genes. Definitions: DCYTB, duodenal cytochrome b; DMT1, divalent metal transporter 1; FTH, ferritin heavy chain; FTL, ferritin light chain; FPN, ferroportin. Data are expressed as mean ± SEM, *n* = 6, different from control, * *p* < 0.05 and ** *p* < 0.01.

Figure 3 provides the effects of Dex administration on duodenal expression of iron metabolism-related genes in rats. No significant difference was found in duodenum iron content between the two groups (Figure 3A). The duodenal iron-metabolism genes including duodenal cytochrome b (*Dcytb*), ferritin light chain (*Ftl*) and ferroportin (*Fpn*) were significantly up-regulated on an mRNA level after consecutive injection with Dex (Figure 3B). As shown in Figure 3D,E, the duodenal DCYTB (*p* = 0.00) and divalent metal transporter 1 (DMT1) (*p* = 0.04) protein expression was significantly decreased, while FPN protein markedly increased (*p* = 0.04) in the Dex group.

### 3.5. Histological Characteristics, Hepatic Iron Content and Hepatic Iron-Metabolism Genes Expression

**Figure 4:**
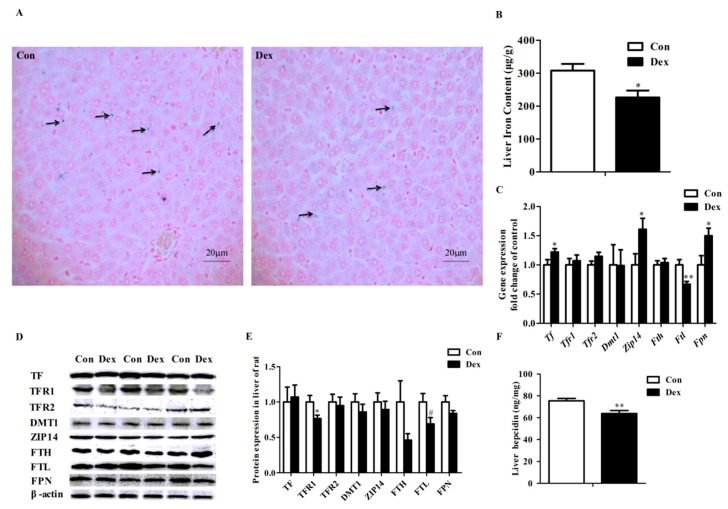
Effects of Dex administration on iron content and iron metabolism genes expression of the liver in rats. (**A**) Hepatic iron detection using the Prussian Blue staining. More iron (blue) was revealed in the rat of the control group. Nuclei are stained with fast red, and cytoplasm are stained pale pink; (**B**) Dex administration reduces hepatic iron content; (**C**) Effect of long-term Dex exposure on hepatic iron-metabolism genes expression; (**D**) Western blot bands of hepatic iron-metabolism genes; (**E**) Hepatic protein expression of iron metabolism-related genes; (**F**) Dex administration decreases liver hepcidin level. Definitions: TF, transferrin; TFR1, transferrin receptor 1; TFR2, transferrin receptor 2; ZIP14, ZRT/IRT-like protein 14. Data are expressed as mean ± SEM, *n* = 6, different from control, * *p* < 0.05, ** *p* < 0.01 and ^#^ *p* = 0.08.

Perls’ Prussian Blue staining of liver sections showed that hepatic cells have least iron density in the Dex exposure group (Figure 4A). Dex administration markedly reduced the iron content in the liver (*p* = 0.02, Figure 4B). Dex injection did not affect hepatic *Tfr1* mRNA abundance (Figure 4C), whereas it did significantly lower the TFR1 protein level (*p* = 0.03) and hepatic hepcidin content (*p* = 0.00) (Figure 4D–F). Reduced expression of both mRNA (*p* = 0.01, Figure 4C) and protein (*p* = 0.08, Figure 4D,E) levels of FTL was observed in rats treated with Dex. No significant changes were detected for these proteins including transferrin (TF), transferrin receptor 2 (TFR2), DMT1, ZRT/IRT-like protein 14 (ZIP14), ferritin heavy chain (FTH) and FPN in the liver of rats after exposure to Dex (Figure 4D,E). In addition, no significant difference was observed in the iron content of the spleen, kidney and dorsal muscles of rats treated with Dex injection over 21 days (data not provided).

### 3.6. Transcription and Post-Transcriptional Regulation of Hepatic Tfr1

**Figure 5:**
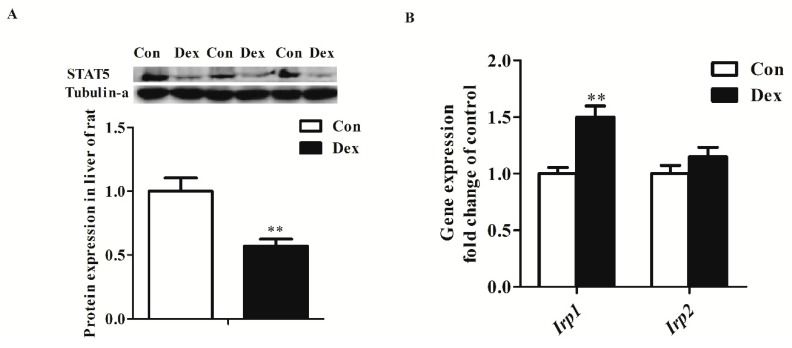

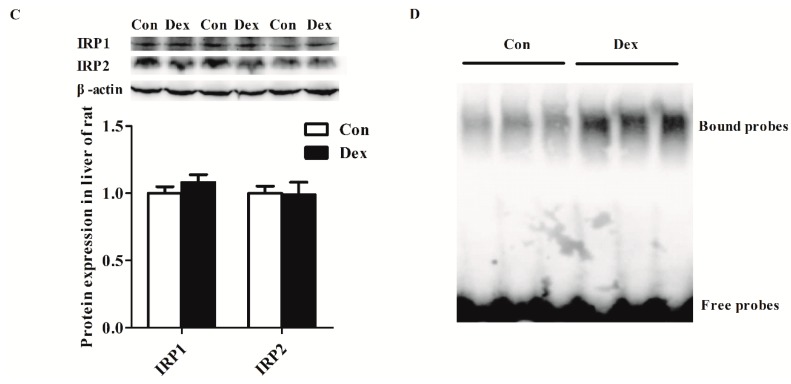
Effects of Dex administration on IRP1/2 expression and IRP–IRE binding activity of *Tfr1* in the liver of rats. (**A**) Western blot bands and protein expression for STAT5; (**B**) Hepatic mRNA abundance of Irp1 and Irp2; (**C**) Western blot bands and protein expression for IRP1 and IRP2; (**D**) Dex administration increases IRP–IRE binding activity of *Tfr1* in the liver of rats. The existence of IRP–IRE binding activity of *Tfr1* was observed through RNA electrophoretic mobility shift assay (REMSA). Definitions: IRP1, iron-regulatory protein 1; IRP2, iron-regulatory protein 2; STAT5, Signal transducers and activators of transcription 5. Data are expressed by means ± SEM, *n* = 6, different from control, ** *p* < 0.01.

Dex administration significantly decreased the *Tfr1* transcription factor for the signal transducers and activators of transcription 5 (STAT5) protein level (*p* = 0.00) (Figure 5A). The IRP/IRE regulatory network is the most common post-transcriptional regulation of Tfr1 expression. Dex administration markedly increased *Irp1* mRNA expression (*p* = 0.00), but did not affect IRP1 and IRP2 protein levels (Figure 5B,C). Nevertheless, the activity of IRP1/2 binding to *Tfr1* mRNA exhibited an obvious increase in Dex-treated rats (Figure 5D).

## 4. Discussion

Stress affects immune function and food intake in a bidirectional way, mainly depending on the intensity and duration of stressors [18,23,29]. Chronic or long-term stress induces suppression of immune function by decreasing immune cell numbers/function and increasing active immunosuppressive mechanisms [18]. In this study, long-term Dex administration reduced the white blood cell counts, lymphocyte counts, and serum IL-6 level, which are the characteristics of immunosuppression. In addition, similar hyper-suppression was observed that the lowered serum glucocorticoid (CORT) level was caused by the feedback inhibition of the HPA axis response to a lower dose of Dex exposure [30,31]. It is also a known fact that stress severity is directly related to reduced food intake and body weight in rodents [29]. Similar to previous studies [23,32,33,34], long-term Dex administration (0.1 mg/kg/day) could inhibit food intake and reduce body weight of female rats fed the less palatable diet in this study. However, the 7-day or 14-day psychological stress exposure induced by the communication box did not affect food intake and body weight [13,35,36]. Long-term and severe stress results in inhibition of growth and food intake, in part because the increasing release of leptin functions as feeding reductions and enhances energy output [23,37,38].

It is revealed that psychological stress reduces blood iron levels [12,13,14,39]. In contrast to the previous findings, our results showed that long-term Dex administration resulted in enhanced serum iron levels in rats. The difference could be induced by stress related to the intensity and duration. DCYTB is a ferric reductase that reduces ferric to ferrous iron [40], then ferrous iron is mainly transported from lumen into the enterocytes by DMT1 in the duodenum [41,42]. FPN is the only known mammalian iron exporter, and transports iron across the basolateral membrane into the circulation [6,43]. It is reported that psychological stress does not affect iron intake, but inhibits iron transport via decreased duodenal FPN expression [14,15]. However, long-term Dex exposure decreases the iron intake, which is closely associated with reduced duodenal DCYTB and DMT1 expression. FPN is down-regulated by hepcidin via proteasome internalization and degradation in enterocytes, macrophages, and hepatocytes [44]. In our results, lowered hepcidin plays a role in increased duodenal FPN expression.

The liver plays a vital role in maintaining systemic iron balance by acting as an exchangeable iron pool. Hepcidin is mainly produced and secreted by hepatocytes [45], which induces degradation of FPN to regulate systemic iron homeostasis. It is proved that hepcidin synthesis is up-regulated by IL-6 through STAT3 signaling pathway [46,47]. The psychological stress could up-regulate expressions of IL-6 and hepcidin [14]. Nevertheless, long-term Dex exposure down-regulated IL-6 level and resulted in reduced hepatic hepcidin content. Liver iron storage is controlled by regulating hepatic iron uptake and release. The mechanisms of iron uptake in the liver are through TFR-dependent pathways (TFR1 and TFR2) and TFR-independent (DMT1 and ZIP14) pathways, respectively [48,49]. Transferrin-bound iron is taken up through a TFR1/2-mediated endocytosis [50,51], whereas non-transferrin-bound iron is transported via DMT1 and ZIP14. FPN is the only known iron exporter, which releases iron from ferritin in animals [6]. Psychological stress increased hepcidin expression and led to hepatic FPN degradation, whereas it develops hepatic iron accumulation in rodents [14]. In this study, other proteins related to iron uptake (TFR2, DMT1, ZIP14) and iron exporter (FPN) expression did not differ in rats treated with Dex exposure. Therefore, reduced liver iron content is closely associated with impaired hepatic iron uptake via a TFR1-dependent pathway.

Tfr1 expression is regulated at transcriptional and post-transcriptional levels. It has been reported that the signal transducer and activator of transcription 5 (STAT5) is an important transcriptional regulator of *Tfr1*, and its mutation causes microcytic anemia associated with severe iron deficiency in mice [52]. Endogenous glucocorticoid exposure generally suppresses growth hormone (GH) release and impairs GH-dependent activation of the transcription factor STAT5 in the liver [52,53,54]. On the other hand, *Tfr1* expression is mainly regulated post-transcriptionally by cellular iron levels through the IRP–IRE system [55]. When cellular iron is deficient, IRPs bind to IREs of *Tfr1* mRNA 3′ UTR and decrease its mRNA turnover and degradation [56]. Previous studies have shown, that a certain degree of psychological stress or glucocorticoid exposure up-regulates *Tfr1* expression, through increasing IRP1 and STAT5 levels to cause liver iron accumulation. In the present study, long-term Dex exposure had no impact on liver *Tfr1* expression, but did supress the hepatic STAT5 protein level and enhanced the IRP–IRE activity of hepatic *Tfr1* in rats. Impaired STAT5 protein expression and increased IRP–IRE activity of hepatic Tfr1 could be jointly responsible for the unchanged *Tfr1* mRNA level.

It has been demonstrated that accelerated protein degradation is induced by glucocorticoids (dexamethasone or cortisol) in vitro [57,58] or in vivo [59]. In eukaryotic cells, ubiquitin–proteasome system and autophagy are the two major pathways that degrade most cellular proteins [60]. Mi et al. (2017) reported that the reduction in COL1A1 protein abundance induced by cortisol was associated with lysosome-mediated autophagic degradation of COL1A1 in fibroblasts [58]. In addition, the stimulatory glucocorticoid increases MyoD degradation via ubiquitin-proteasome-dependent proteolysis [59]. Therefore, the lysosome-mediated autophagic degradation and/or ubiquitin-dependent proteolysis may play a role in the down-regulation of hepatic TFR1 protein abundance at the post-translational level.

In summary, long-term Dex exposure disturbs iron homeostasis and mainly results in enhanced serum iron level, suppressed TFR1 protein expression and decreased hepatic iron content. The decreasing liver iron content is closely associated with the inhibition of hepatic iron uptake through a TFR1-mediated pathway.

## Figures and Tables

**Table 1:** Nucleotide sequences of specific primers.

Target Genes	Sequences (5′ to 3′)		Products	GenBank No.
*Dcytb*	F:agacttggacgaggatact	R:ggcagaccaggatatgtg	104 bp	NM_001011954.1
*Dmt1*	F:tcacttggtcctcgttct	R:tcactaacagcctccttatag	139 bp	NM_013173.2
*Fth*	F:gtcactactggaacttcaca	R:ttcaggtaatgcgtctcaat	216 bp	NM_012848.2
*Ftl*	F:gcagaagccatctcaaga	R:ttccaagaagtcacagagg	197 bp	NM_022500.4
*Fpn*	F:aggaaggatgctgtggat	R:tgtcaagaggaggctgtt	115 bp	NM_133315.2
*Tf*	F:atcagactccagcatcaac	R:ccaatacacaggtcacaga	198 bp	NM_001013110.1
*Tfr1*	F:cacttacggtcagcactt	R:cacaactcactggacttaga	114 bp	NM_022712.1
*Tfr2*	F:gttggtggttggtgaaga	R:acatagtgcgtgtcagtc	240 bp	NM_001105916.1
*Zip14*	F:ttggaagaagcactgagag	R:ttggaagaagcactgagag	149 bp	NM_001107275.1
*Irp1*	F:cgatgctgtgaagaagttg	R:aatgaacctggatggaatga	92 bp	NM_017321.1
*Irp2*	F:ggcacagattctcatataacc	R:tcacatccaaccacctct	131 bp	NM_022863.2
*Arbp*	F:tagagggtgtccgcaatgtg	R:cagtgggaaggtgtagtcagtc	217 bp	NM_022402.2

Note: *Dcytb*, duodenal cytochrome b; *Dmt1*, divalent metal transporter 1; *Fth*, ferritin heavy chain; *Ftl*, ferritin light chain; *Fpn*, ferroportin; *Tf*, transferrin; *Tfr1*, transferrin receptor 1; *Tfr2*, transferrin receptor 2; *Zip14*, ZRT/IRT-like protein 14; *Irp1*, iron regulatory protein 1; *Irp2*, iron regulatory protein 2; *Arbp*, acidic ribosomal phosphoprotein P0.

**Table 2:** Administration of Dex on serum iron parameters in rats.

Parameters	Con	Dex	P-Value
Serum iron (µmol/L)	45.29 ± 2.12	55.09 ± 2.80	0.01
UIBC (µmol/L)	37.19 ± 2.02	37.24 ± 4.10	0.20
TIBC (µmol/L)	82.48 ± 2.06	92.33 ± 3.44	0.20
Transferrin (µg/dL)	131.40 ± 2.64	135.20 ± 3.21	0.37
TS (%)	52.59 ± 2.82	60.28 ± 3.53	0.11
Ferritin (ng/mL)	6.85 ± 0.34	7.34 ± 0.80	0.60
sTfR (nmol/L)	32.29 ± 8.10	135.42 ± 38.96	0.03

Note: sTfR, souble transferrin receptor; Tf, transferrin; TS, transferrin saturation; TIBC, total iron binding capacity; UIBC, unsaturated iron binding capacity. Data are expressed as mean ± SEM, *n* = 10.
