# Erratum: Long-Term Dexamethasone Exposure Down-Regulates Hepatic TFR1 and Reduces Liver Iron Concentration in Rats; *Nutrients* 2017, *9*, 617

**DOI:** 10.3390/nu10040467

**Published:** 2018-04-10

**Authors:** Nutrients Editorial Office

**Affiliations:** MDPI, St. Alban-Anlage 66, 4052 Basel, Switzerland; nutrients@mdpi.com

The Editorial Office of *Nutrients* would like to report an error in the published paper [1]. The affiliation of the authors should be changed from “Key Laboratory of Animal Physiology and Biochemistry, College of Veterinary Medicine, Agricultural University, Nanjing 210095, China” to “Key Laboratory of Animal Physiology and Biochemistry, College of Veterinary Medicine, Nanjing Agricultural University, Nanjing 210095, China”. 

We apologize for any inconvenience caused to the readers by this change. The change does not affect the scientific results. The manuscript will be updated and the original will remain available on the article webpage.
